# No Effect of Acute Eccentric Resistance Exercise on Immune Responses to Influenza Vaccination in Older Adults: A Randomized Control Trial

**DOI:** 10.3389/fphys.2021.713183

**Published:** 2021-08-12

**Authors:** Mahmoud T. Elzayat, Melissa M. Markofski, Richard J. Simpson, Mitzi Laughlin, Emily C. LaVoy

**Affiliations:** ^1^Department of Health and Human Performance, University of Houston, Houston, TX, United States; ^2^Department of Nutritional Sciences, College of Agriculture and Life Sciences, University of Arizona, Tucson, AZ, United States; ^3^Fondren Orthopedic Research Institute, Houston, TX, United States

**Keywords:** immunity, virus, exercise, aging, vaccine

## Abstract

**Introduction:**

Older adults are at elevated risk for morbidity and mortality caused by influenza. Vaccination is the primary means of prophylaxis, but protection is often compromised in older adults. As resistance exercise mobilizes immune cells into muscle, it may enhance vaccination response.

**Purpose:**

Compare antibody and cell mediated immune responses to influenza vaccination in older adults who performed eccentric resistance exercise immediately prior to vaccination to those who did not exercise.

**Methods:**

Twenty nine resistance training-naive older adults (20 women, 73.9 ± 5.3 years) were randomized to 1 of 3 groups: vaccination in the same arm that exercised (Ex-S), vaccination in the opposite arm that exercised (Ex-Op), and seated rest (No-Ex). Exercise consisted of 10 sets of 5 eccentric unilateral repetitions at 80% of the pre-determined concentric one repetition maximum. Lateral raises were alternated with bicep curls. No-Ex sat quietly for 25 min. Following exercise or rest, all received the 2018 quadrivalent influenza vaccine (Seqirus Afluria) in the non-dominant deltoid. Antibody titers against each influenza vaccine strain were determined by hemagglutinin inhibition assays at baseline, 6-, and 24-weeks post-vaccination. Influenza-specific T cells were quantified after stimulation with the vaccine by intracellular cytokine staining.

**Results:**

No significant group x time effects were found in antibody responses to any strain (interaction for A/H1N1: *p* = 0.682; A/H3N2: *p* = 0.644; B/Colorado/06/2017: *p* = 0.262; B/Phuket/3073/2013: *p* = 0.851). Groups did not differ in fold-increase of antibody titers 6- and 24-weeks post-vaccination. Influenza-specific T-cells did not differ between groups at any time (comparison at baseline: *p* = 0.985; 6-weeks: *p* = 0.889; 24 weeks: *p* = 0.857). One subject (Ex-S) reported flu-like symptoms 18 weeks post-vaccination.

**Conclusion:**

Acute arm eccentric exercise did not influence antibody titers or cell mediated immune responses to the influenza vaccine delivered post-exercise in older adults. More strenuous exercise may be required for exercise to act as an adjuvant. ClinicalTrials.gov Identifier: NCT03736759.

## Introduction

The aging immune system leaves older adults more vulnerable to infections, including with influenza virus. Influenza infection is associated with the death of thousands of people in the United States each year, approximately 90% of which occur among older adults (≥65 years) (Thompson et al., 2003, 2004, 2009). The primary means of protection against influenza is through annual vaccination with the seasonal influenza vaccine. Unfortunately, protective responses are impaired in this population, which likely accounts for the increased prevalence of infection in older adults (Goodwin et al., 2006; Song et al., 2010; Castilla et al., 2013). One common approach to assess protection is measurement of influenza-specific antibody titers. Older adults exhibit lower antibody responses to the influenza vaccine leading to a clinical vaccine efficacy of just 17–53%, compared to 70–90% efficacy in younger adults (Goodwin et al., 2006; Castilla et al., 2013). Furthermore, antibody titers to influenza may decline more rapidly in older adults, meaning the effectiveness of the influenza vaccine may be further reduced if influenza exposure occurs late in the season (Song et al., 2010). Cell mediated immunity provided by influenza-specific cytotoxic T-cells also offers protection against disease, maybe induced by vaccination (Ennis et al., 1981; Sridhar et al., 2013). Unfortunately, impaired T-cell responses after influenza vaccination have been reported in older adults relative to younger adults (Fagiolo et al., 1993). This reduction in vaccination response is present even in older adults without chronic disease (Fagiolo et al., 1993; Hainz et al., 2005; Van Epps et al., 2017).

Considering the importance of generating protective immune responses against influenza infection, the development of strategies to improve vaccine responses among older adults is vital. Deficits in the antibody response to vaccines arise from age-related declines in the function of B-cells and T-cells (Haynes and Swain, 2006; Siegrist and Aspinall, 2009), and potentially, antigen-presenting cells including dendritic cells (Shurin et al., 2007). Different formulations of the influenza vaccine for older adults have been proposed to enhance the protective levels of immune responses, such as the use of different adjuvants, different modes of administration, and/or increased antigen dose in the vaccine (Banzhoff et al., 2003; Keitel et al., 2006; Leroux-Roels et al., 2007; Della Cioppa et al., 2014). Although these strategies are typically more immunogenic than conventional non-adjuvanted influenza vaccines, they are also associated with greater injection site symptoms (e.g., erythema, swelling, and pain) and general systemic symptoms (e.g., malaise) post-vaccination (Banzhoff et al., 2003; Keitel et al., 2006; Leroux-Roels et al., 2007; Della Cioppa et al., 2014). While the safety profile is clinically acceptable, increases in the potential discomfort of vaccination could reduce vaccine uptake. Further, increases in the antigen dose in certain vaccine formulations require increased manufacturing capabilities and thus may limit available vaccine doses. Thus, there is a need to develop a simple, cost-effective approach with minimal side-effects to enhance influenza vaccine responses in older adults.

One potential method to enhance vaccine responses in older adults may be through localized resistance exercise designed to induce mild transient muscle damage. Eccentric exercise, the application of tension as a muscle lengthens, reliably causes mild muscle damage and a local inflammatory response consisting of increased blood flow, vascular permeability, and immune cell invasion into the targeted muscle (Proske and Morgan, 2001; Peake et al., 2005). The inflammatory response is especially noted in resistance-training naïve individuals (Nosaka et al., 2001; Peake et al., 2005). In young adults, resistance exercise involving eccentric contractions of the deltoid and biceps bracchi muscles immediately prior to inoculation at this site improves antibody titer responses to vaccination (Edwards et al., 2007, 2010, 2012). While a recent study in older adults failed to find an effect of moderate-intensity whole-body resistance training on seroprotection, it may be that this exercise protocol was insufficient at eliciting an immune response (Bohn-Goldbaum et al., 2020). A strategy employing acute eccentric resistance exercise targeting the inoculation site has yet to be implemented in older adults who are more likely to benefit from these adjuvant effects of exercise. Moreover, it has not been determined from these published studies if the exercise effects are due to a local or a systemic response, as only the exercised arm has been inoculated. Eccentric resistance exercise also causes change in systemic immune parameters, such as increases in circulating leukocytes, altered expression of migration and adhesion molecules on neutrophils and monocytes, and increases in pro-inflammatory cytokines (Peake et al., 2005). Therefore, exercise could act as an adjuvant through generalized immune upregulation.

The aim of this study was to determine the impact of a single bout of unaccustomed eccentric arm/shoulder resistance exercises performed immediately prior to inoculation on antibody and cell mediated immune responses to the seasonal influenza vaccine in older adults. We also aimed to compare the effect of identical bouts of exercise prior to vaccination in the inoculated arm and the non-inoculated arm. We hypothesized that vaccination in the arm that performed the eccentric resistance exercise would increase antibody titers and influenza-specific T-cells at 6 and 24 weeks post-vaccination relative to no-exercise and vaccination in the arm that did not exercise. These experiments will allow us to determine if a single bout of eccentric resistance exercise can be used as a simple and inexpensive vaccine adjuvant, with low side-effects, in community dwelling older adults. These experiments will also help differentiate between the local and systemic effects of eccentric exercise on vaccine enhancement.

## Materials and Methods

### Study Design and Participants

This parallel randomized controlled trial (ClinicalTrials.gov Identifier: NCT03736759) compared vaccine responses at three time points across three groups. Data were collected October 2018 through April 2019 in Houston, TX, United States. Men and women aged ≥ 65 years were recruited from the metropolitan region of Houston, TX. Participants were screened to ensure they were non-frail (screened using Fried’s criteria; Fried et al., 2001), non-smokers (>10 years), were not institutionalized, and met the American College of Sports Medicine criteria for participation in exercise (American College of Sports Medicine, et al., 2005). Participants were excluded if they reported: (i) a history of immune disease or vaccine-related allergies, (ii) having a physician-confirmed influenza infection in the prior year, (iii) regular use of medications known to affect the immune system, (iv) were bedridden in the prior 3 months, (v) engagement in resistance arm exercises in the prior 6 months, (vi) an impairment limiting exercise or prohibiting informed consent, and vii) already vaccinated with the 2018 seasonal influenza vaccine. This population was expected to manifest age-attenuated responses to vaccination but be able to safely complete the bout of eccentric exercise. All participants were naïve to resistance training of the upper body to preclude the repeated bout effect potentially minimizing inflammatory responses (Nosaka et al., 2001). This study was approved by the Institutional Review Board at the University of Houston (STUDY00000542) and was carried out in accordance with the Code of Ethics of the World Medical Association (Declaration of Helsinki).

**FIGURE 1:**
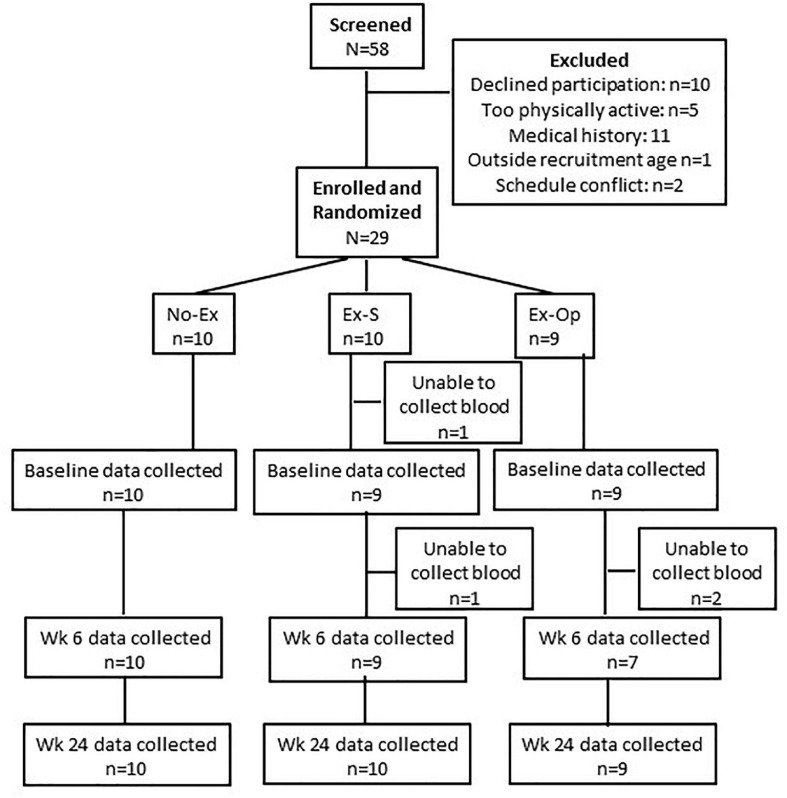
CONSORT flow diagram.

Fifty-eight participants were initially screened for eligibility by phone or email; 16 did not meet inclusion criteria, and 12 declined to participate further or had schedule conflicts (Figure 1). Thirty participants were further screened in-person during the beginning of the first visit. Screening included a blood-pressure measurement, a survey of health behaviors and vaccine questions, the Mini-Mental State Examination, Fried’s Frailty Criteria (including handgrip strength determination using a hydraulic hand dynamometer), and the ACSM/AHA Exercise Readiness Questionnaire. One participant was excluded from further participation due to exclusion criteria. The nature of the study and possible consequences of participation were discussed with each participant, and 29 participants provided informed consent. Participants were randomly assigned to one of three groups at a 1:1:1 ratio, stratified by sex: (Thompson et al., 2004) non-exercise inoculated control (No-Ex); (Thompson et al., 2003) vaccination in the same arm that performed eccentric exercise of the deltoid and biceps bracchi (Ex-S); and (Thompson et al., 2009) vaccination in the opposite arm that performed eccentric exercise of the deltoid and biceps bracchi (Ex-Op). Randomization was achieved via a random number string generated in Microsoft Excel following the recommendations for adaptive randomization by Hoare et al. (2013). All participants completed Visit 1 in October 2018.

Participants completed two additional visits 6- and 24-weeks post-vaccination to provide a blood sample. These visits were expected to correspond to peak antibody-responses (6-weeks post-vaccination) (Rastogi et al., 1995) and also indicate if protection was provided for the entire influenza season (24-weeks post-vaccination). Participants were compensated for their participation in each of the three visits with a gift-card.

### Intervention and Vaccination

Following screening and consent process during Visit 1, participants completed questionnaires relating to life-stress and health behaviors (Perceived Stress Scale (Cohen et al., 1983), General Health Questionnaire-28 (Goldberg, 1978), RAND 36-Item Health Survey (Hays et al., 1993), and a venous blood sample from the arm.

Participants assigned to Ex-S and Ex-Op completed single arm estimated 1 repetition maximum (1RM) tests for the bicep curl and lateral raise exercises. Briefly, participants completed 8–10 unassisted repetitions of the lateral raise exercise (shoulder abduction to 90° with elbow extension) and bicep curl exercise (elbow flexion) using dumb bells of increasing weight until 8 repetitions could not be completed with proper technique. The 1RM for each exercise was estimated using the Brzycki Formula (Brzycki, 1993).

After the experimental resistance exercise load was determined, participants completed 10 sets of 5 repetitions of the eccentric component of each movement at 80% of their estimated 1RM by lowering the dumbbell in a controlled manner over the course of 4 s. A study team member assisted with the concentric component of the exercise. Each experimental set consisted of one set of lateral raise exercise alternated with one set of bicep curl exercise. Weight was lowered if technique suffered to ensure that participants could complete the same number of repetitions. Participants rested 15 s after each set of lateral raise exercise (intra-set rest) and 30 s after each set of bicep curl exercise (inter-set rest). Participants in Ex-S completed the exercises in their non-dominant arm whereas Ex-Op completed the exercises in their dominant arm. Testing and exercise were completed in approximately 25 min. No-Ex participants rested quietly for 25 min.

Following exercise or seated rest, all participants were provided the quadrivalent influenza vaccine developed for the 2018–2019 influenza season (Afluria quadrivalent influenza vaccine; Seqirus; Lot 02544611A). The quadrivalent vaccine included four virus strains: A/Michigan/45/2015 (H1N1), A/Singapore/INFIMH-16-0019/2016 (H3N2), B/Colorado/06/2017, and B/Phuket/3073/2013. Injections were given intramuscularly, using a 25 gauge 25 mm needle, directly into the deltoid of the non-dominant arm.

Participants were asked to report muscle soreness level using a visual analog scale before and after the injection, as well as each day for the next 7 days after injection via a scheduled phone call with a study team member (secondary outcome). Participants were contacted monthly for the remainder of the 6-month intervention to survey for flu-like symptoms (secondary outcome) and were asked to contact the study team if diagnosed with influenza.

### Hemagglutinin Inhibition Assays

A primary outcome of this study was antibody titers to each influenza strain included in the vaccine. Venous blood was collected into 10 mL serum collection tubes (Vacutainer, BD) prior to vaccination (Baseline), 6 weeks-post vaccination, and 24 weeks-post vaccination. Serum was isolated by centrifugation from each sample within 2 h of blood draw, and stored at −80°C. Following the final blood collection, all samples were shipped overnight on dry ice to a commercial laboratory (Southern Research, Birmingham, AL) for the measurement of anti-influenza antibodies against the four influenza virus strains present in the vaccine. A standard microtiter hemagglutination inhibition assay was performed by researchers blinded to the intervention. Samples were analyzed in duplicate with a repeat performed as per standard operating procedure. Correctness of data was verified by an independent operator who did not perform the assay.

Fold-increase (post-vaccine titer/pre-vaccine titer), seroprotection (antibody titer ≥40), and seroconversion (≥4-fold increase) were calculated from the geometric mean antibody titers (Beyer et al., 2004).

### Cell Mediated Immunity Assays

A second key outcome of this study was influenza-specific T-cells. Venous blood was collected into 10 mL collection tubes treated with Sodium Heparin (Vacutainer, BD) prior to vaccination, 6 weeks-post vaccination, and 24 weeks-post vaccination. Within 2 h of collection, peripheral blood mononuclear cells (PBMCs) were isolated using density gradient centrifugation (Histopaque-1077, Sigma) and cryopreserved at −80°C for later bulk analyses. On experiment day, PBMCs were thawed and incubated at a concentration of 1 × 10^6^ cells/ml in 5%FBS-RPMI (Gibco) at 37° with 5% CO_2_. Cells were stimulated for 18 h with phorbol 12-myristate 13-acetate (PMA) and ionomycin, previously titrated vaccine, or RPMI (unstimulated). Monensin (2μM; eBioscience) was added to all cells for the final 6 h. After washing with PBS, cells were labeled with previously titrated fluorescently tagged monoclonal antibodies (all Miltenyi Biotec Inc., Gladbach, Germany): Viogreen-CD3 and APC-CD8. Following washing, cells were fixed and permeabilized using CytoPerm kit (BD Bioscience), and then incubated with PE-anti-IFN-γ. Cells were analyzed with a MACSQuant analyzer flow cytometer (Milteny Biotec Inc.). Compensation beads were used to compensate for spectral overlap in each panel. Single color tubes were used to identify positive and negative staining by each antibody. Lymphocytes were identified by forward and side scatter characteristics and gated electronically using MACSQuantify^TM^ software. IFN-γ expressing cells were assessed within CD3^+^ T-cells. Background levels of IFN-γ expressing cells (present in control condition) were subtracted from experimental and positive controls.

### Statistical Analyses

Data were screened for normality and the presence of outliers graphically and homogeneity of variance was assessed by Levene’s test. Participant characteristics were compared across the three groups by ANOVA; exercise performance measures were compared across the two exercise groups by *t*-tests.

Antibody titers (geometric means) were base-2 logarithmically transformed and normality of residuals was confirmed through examination of P-P plots. Log2 titers were analyzed using random-intercepts maximum likelihood mixed models with a variance components covariance structure. Models included time (three time points), group (three groups), and the interaction of group by time. Age was included as a covariate. Group differences within each timepoint were assessed though pairwise comparisons of estimated marginal means, adjusted for multiple comparisons by the method of Sidak. Fold-change in antibody titer was calculated for each time point relative to baseline titer (6-week titer/baseline titer; 24-week titer/baseline titer) and group-effects were assessed by Kruskal-Wallis Test. Effect sizes are reported as ε^2^ (Tomczak and Tomczak, 2014). Titers against each of the four virus strains were considered separately.

The change in the proportion of IFN-γ T-cells at baseline, 6-, and 24-weeks following stimulation was assessed using Friedman’s test, and Kendall’s W coefficient reported as measure of effect size (Tomczak and Tomczak, 2014). The proportion of IFN-γ T-cells at each time point were compared between the groups using the Kruskal-Wallis Test. All analyses were completed using IBM SPSS Statistics for Windows, Version 26. *p* < 0.05 was accepted as significant.

## Results

### Participants and Exercise

Twenty-nine participants (20 women) were randomized into one of three groups: No-Ex, Ex-S, and Ex-Op. Participant characteristics are shown in Table 1. Groups did not differ in these characteristics.

**TABLE 1:** Participant characteristics.

N (Female)	All 29 (20)	No-Ex 10 (7)	Ex-S 10 (7)	Ex-OP 9 (5)	*p*
Age (years)	73.9 ± 5.3	70.9 ± 5.7	75.8 ± 5.0	75.1 ± 5.3	0.132
Height (cm)	165 ± 6.8	168 ± 8.4	165 ± 7.0	164 ± 4.5	0.525
Weight (kg)	75.0 ± 14.6	75.0 ± 14.6	80.7 ± 16.4	69.7 ± 9.9	0.269
Body mass index (kg/m^2^)	27.3 ± 4.9	26.2 ± 4.6	29.7 ± 5.7	25.8 ± 3.7	0.189
Grip strength dominant (kg)	27.4 ± 8.5	29.9 ± 10.5	27.1 ± 5.7	24.9 ± 8.7	0.450
Grip strength non-dominant (kg)	25.1 ± 6.9	28.9 ± 9.6	23.7 ± 3.7	22.8 ± 5.3	0.123

*Values are mean ± SD; p-value is associated with F-tests comparing No-Ex, Ex-S, and Ex-OP.*

Ex-S and Ex-Op completed a total of 50 repetitions of the lateral raise and bicep curl exercises. In both groups, the weight was lowered from the 80% of 1RM used at the start of the intervention as participants fatigued and technique suffered. Ex-S and Ex-Op did not differ in exercise performance (Table 2).

**TABLE 2:** Resistance exercise performed by Ex-S (exercise in non-dominant arm) and Ex-OP (exercise in dominant arm).

	Ex-S	Ex-OP	*p*
1 RM lateral raise (kg)	2.6 ± 0.8	2.9 ± 0.7	0.471
1 RM bicep curl (kg)	4.2 ± 1.2	4.2 ± 2.1	0.715
Intervention lateral raise (average of 50 rep) (kg)	1.9 ± 0.7	2.2 ± 0.7	0.400
Intervention lateral raise (average of 50 rep) (%1RM)	72.5 ± 11.0	75.9 ± 8.1	0.796
Intervention bicep curl (average of 50 rep) (kg)	3.1 ± 0.7	3.3 ± 1.9	0.791
Intervention bicep curl (average of 50 rep) (%1RM)	75.9 ± 9.6	76.5 ± 5.6	0.862

*Values are mean ± SD; p-value is associated with t-tests comparing Ex-S and Ex-OP. 1RM: 1 repetition maximum estimated using Brzycki Formula; intervention weight is the average weight used across the 10 sets of 5 repetitions.*

Participants were asked to report the presence of any soreness in the vaccinated and unvaccinated arms for each of 7 days post-vaccination. Eight of 29 participants (3 in No-Ex, 3 in Ex-S, and 2 in Ex-Op) reported soreness in the vaccinated arm on at least 1 day; all reports of soreness resolved within 6 days. One participant in Ex-Op also reported soreness in the non-vaccinated arm (the arm that exercised); this soreness also resolved within 6 days.

### Antibody Titer

**FIGURE 2:**
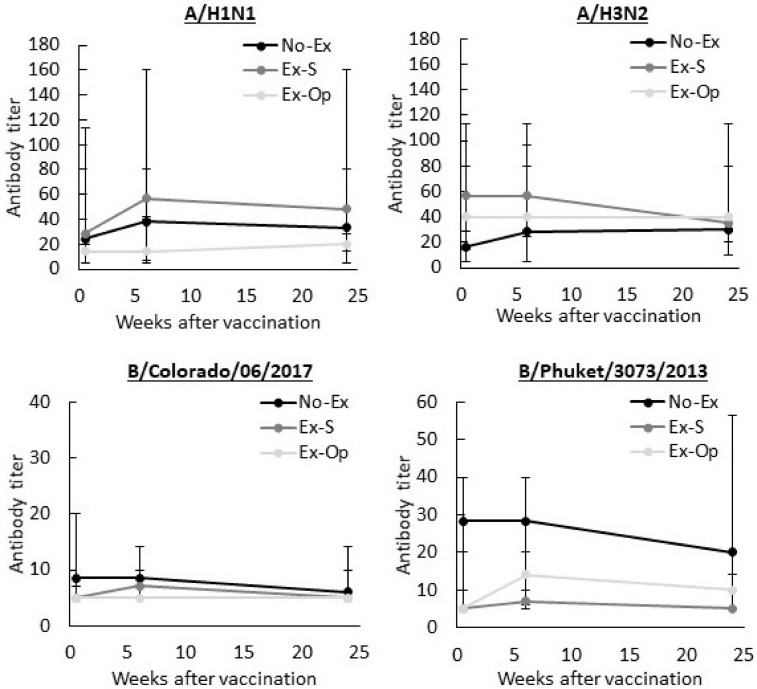
Influenza-specific antibody titers before, 6-, and 24-weeks after influenza vaccination. Median and interquartile range of the geometric mean antibody titers for each strain shown. No-Ex, control group; Ex-S, exercise in inoculated arm; Ex-Op, exercise in arm not inoculated.

Serum collected at baseline, 6-, and 24-weeks post-vaccination was assessed by hemagglutinin inhibition assays to determine antibody titers to the four influenza strains included in the vaccine. Geometric mean antibody titers are displayed in Figure 2. No-Ex, Ex-S, and Ex-Op did not differ in antibody titer to any strain across the three time points: A/H1N1, *F*(4, 50.56) = 0.575, *p* = 0.682; A/H3N2, *F*(4, 50.57) = 0.629, *p* = 0.644; B/Colorado/06/2017, *F*(4, 51.37) = 1.355, *p* = 0.262; and B/Phuket/3073/2013, *F*(4, 50.73) = 0.338, *p* = 0.851. There was also no difference in antibody titer between the three visits, regardless of group assignment: A/H1N1, *F*(2, 50.53) = 0.726, *p* = 0.489; A/H3N2, *F*(2, 50.52) = 1.869, *p* = 0.165; B/Colorado/06/2017, *F*(2, 51.31) = 1.533, *p* = 0.226; and B/Phuket/3073/2013, *F*(2, 50.69) = 2.693, *p* = 0.077. Accordingly, rates of seroprotection (antibody titer ≥ 40) remained low, particularly for B/Colorado/06/2017 (Table 3).

**TABLE 3:** Number and proportion of participants overall and within group exhibiting seroprotection and seroconversion 6- and 24-weeks post-vaccination.

		At 6-weeks		At 24-weeks	
		Seroprotection	Seroconversion	Seroprotection	Seroconversion

	Antigen	n (%)	n (%)	n (%)	n (%)
All	A/H1N1	14 (53.8)	4 (26.0)	13 (44.8)	2 (7.1)
	A/H3N2	15 (57.7)	3 (12.0)	16 (55.1)	3 (10.7)
	B/Colorado/06/2017	0 (0.0)	2 (8.0)	2 (6.9)	1 (3.6)
	B/Phuket/3073/2013	6 (23.0)	3 (12.0)	5 (17.2)	2 (7.1)
No-Ex	A/H1N1	5 (50.0)	1 (10.0)	5 (50.0)	0 (0.0)
	A/H3N2	4 (40.0)	2 (20.0)	5 (50.0)	2 (20.0)
	B/Colorado/06/2017	0 (0.0)	0 (0.0)	2 (20.0)	1 (10.0)
	B/Phuket/3073/2013	4 (40.0)	1 (10.0)	4 (40.0)	1 (10.0)
Ex-S	A/H1N1	7 (77.8)	2 (22.2)	6 (60.0)	1 (10.0)
	A/H3N2	7 (77.8)	1 (11.1)	5 (50.0)	1 (10.0)
	B/Colorado/06/2017	0 (0.0)	2 (22.2)	0 (0.0)	0 (0.0)
	B/Phuket/3073/2013	2 (22.2)	1 (11.1)	1 (10.0)	0 (0.0)
Ex-OP	A/H1N1	2 (28.6)	1 (14.3)	2 (22.2)	1 (11.1)
	A/H3N2	4 (57.1)	0 (0.0)	6 (66.7)	0 (0.0)
	B/Colorado/06/2017	0 (0.0)	0 (0.0)	0 (0.0)	0 (0.0)
	B/Phuket/3073/2013	0 (0.0)	1 (14.3)	0 (0.0)	1 (11.1)

*Seroprotection: antibody titer ≥ 40; seroconversion: fold-change from pre-vaccination ≥ 4.*

**FIGURE 3:**
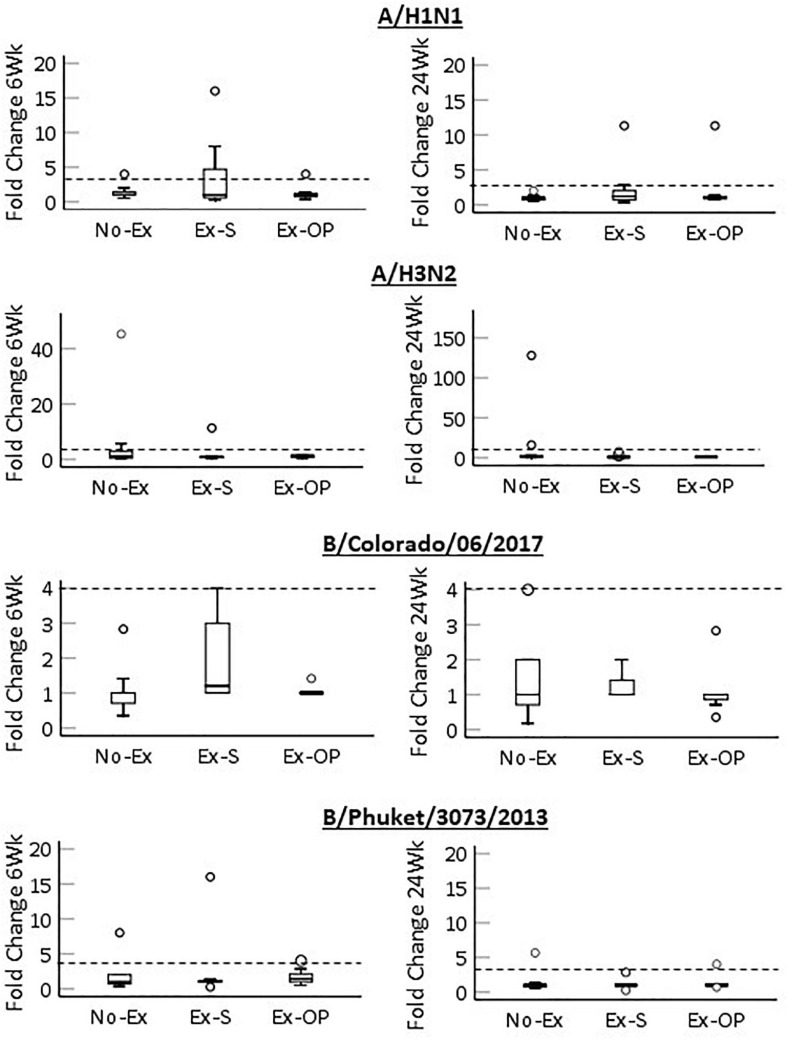
Fold change in antibody titer from baseline 6-weeks post-vaccination **(left)** and 24-weeks post-vaccination **(right)** for each strain by group. Median (black bar), 1st and 3rd quartiles (box), confidence interval (error bar) and outliers (circles) are shown. Dashed line indicates seroconversion.

To understand if groups differed in the change in antibody titer from baseline, we compared fold-change at 6- and 24-weeks between groups (Figure 3). Overall, rates of seroconversion (fold-change ≥ 4) were low (Table 3). No group differences in 6- or 24-week fold change were noted for any virus strain (Table 4), and most effect sizes were small. However, a moderate effect size was noted for fold-change at 6-weeks for B/Colorado/06/2017 (ε^2^ = 0.169). Visual inspection of the data suggests a trend for a greater fold-change in Ex-S (Figure 3). Mean fold increases at 6-weeks in response to B/Colorado/06/2017 were 1.08 (No-Ex), 1.93 (Ex-S), and 1.06 (Ex-Op).

**TABLE 4:** Effect of group on fold-change in antibody titers 6- and 24-weeks post-vaccination.

Antigen	*H*	*p*	ε^2^
**Fold-change 6-weeks**			
A/H1N1	0.162	0.922	0.007
A/H3N2	0.738	0.692	0.032
B/Colorado/06/2017	4.512	0.105	0.169
B/Phuket/3073/2013	0.734	0.693	0.032
**Fold-change 24-weeks**			
A/H1N1	0.625	0.732	0.024
A/H3N2	2.072	0.355	0.076
B/Colorado/06/2017	1.489	0.475	0.056
B/Phuket/3073/2013	1.232	0.540	0.047

*Fold-change: titer at indicated week/baseline titer, H, p, and ε^2^ from Kruskal-Wallis tests with 2 degrees of freedom.*

Despite the low rates of seroprotection, only one participant (Ex-S) reported flu-like symptoms 18 weeks post-vaccination; influenza infection was not clinically confirmed in this participant.

### Cell Mediated Immunity

**FIGURE 4:**
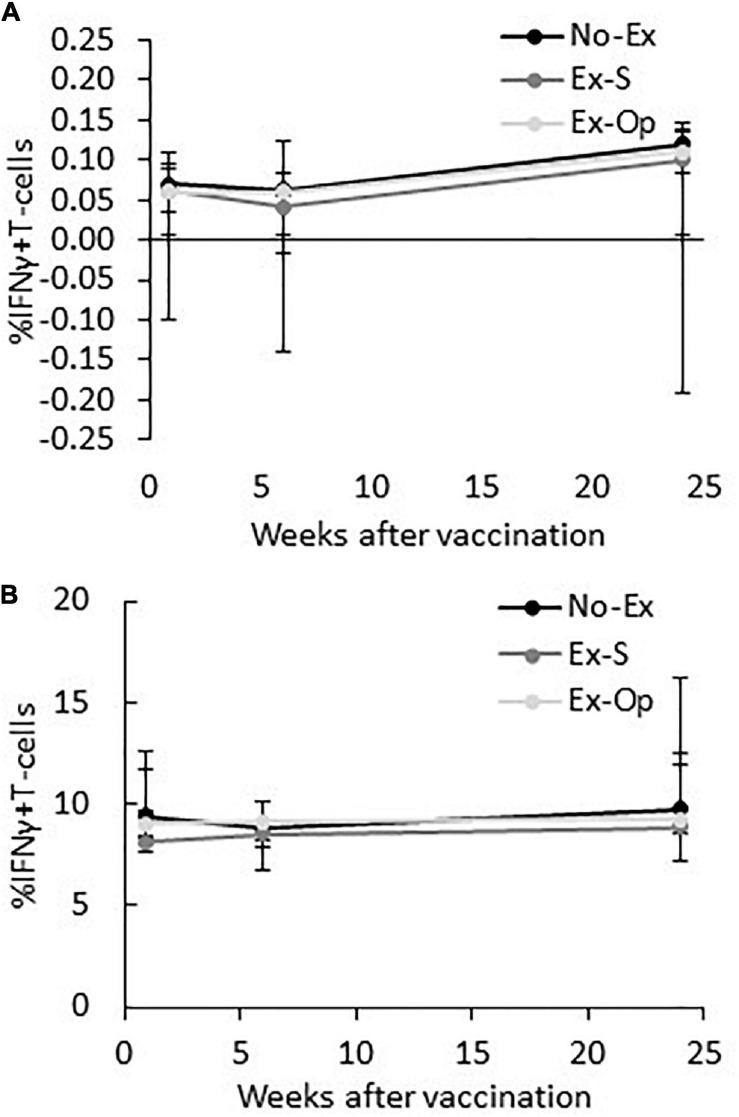
IFN-γ expressing T-cells at baseline, 6-weeks post-vaccination, and 24-weeks post-vaccination by group following stimulation with influenza vaccine **(A)** or PHA **(B)**. Median and interquartile range are shown.

Regardless of group assignment, vaccination did not induce an increase in influenza-specific T-cells and effect sizes remained small [No-Ex: X ^2^(2) = 4.79, *p* = 0.091, *W* = 0.263; Ex-S: X ^2^(2) = 0.26, *p* = 0.878, *W* = 0.167; Ex-Op: X ^2^(2) = 2.48, *p* = 0.289, *W* = 0.111; Figure 4]. Groups did not differ in the proportion of IFN-γ+ T-cells at any time point [baseline: H(2) = 0.29, *p* = 0.985, ε^2^ = 0.001; 6-weeks: H(2) = 0.24, *p* = 0.889, ε^2^ = 0.011; 24-weeks: H(2) = 0.31, *p* = 0.857, ε^2^ = 0.014]. T-cell responses to the positive control remained constant throughout the experiment [No-Ex:X ^2^(2) = 4.75, *p* = 0.093; Ex-S: X ^2^(2) = 3.00, *p* = 0.223; Ex-Op: X ^2^(2) = 2.00, *p* = 0.368]; and did not differ between groups [baseline: H(2) = 1.45, *p* = 0.484; 6-weeks: H(2) = 0.50, *p* = 0.781; 24-weeks: H(2) = 0.30, *p* = 0.859; Figure 4].

## Discussion

We investigated the effect of a single session of eccentric-focused resistance exercise on influenza vaccine responses in older adults. Older adults frequently have insufficient responses to the influenza vaccine and so are at increased risk for influenza infection. We hypothesized that eccentric exercise performed in the same arm subsequently vaccinated would improve protective immune responses to the vaccine. Our outcomes of interest, hemagglutinin inhibition antibody titers and influenza-specific T-cells, were compared between three groups before and after (6- and 24-weeks) vaccination with the seasonal quadrivalent influenza vaccine. Contrary to our hypothesis, no differences were found between No-Ex, Ex-S, and Ex-Op across this timeframe, indicating that an acute bout of unfamiliar eccentric resistance exercise, performed just before vaccination either in the same or opposite arm, did not influence antibody responses to the vaccine in this population of older adults.

Observations that greater physical activity levels are associated with greater immune responses following influenza vaccination have provided a rationale for chronic exercise training interventions as a means to improve vaccine responses (Kohut et al., 2002; Schuler et al., 2003; Whitham and Blannin, 2003; Keylock et al., 2007; Pascoe et al., 2014). Although 5–10 month long exercise training interventions have had moderate success in enhancing anti-influenza antibody titers (Kohut et al., 2004; Yang et al., 2007; Woods et al., 2009), the time commitment required may render such interventions impractical for broad implementation. Single sessions of exercise have also been proposed as a means of enhancing immune responses to vaccination. A moderate intensity aerobic exercise bout performed prior to inoculation yielded higher influenza antibody titers in young women, but not young men, 4- and 20-weeks post-vaccination (Edwards et al., 2006). In a subsequent examination by the same group it was reported that eccentric resistance exercise prior to influenza vaccination also significantly increased antibody titers 6-weeks post-vaccination by approximately 1.5X the control condition in young women, corresponding to a medium effect size (Edwards et al., 2007). Though the current study mirrored the eccentric-focused exercise protocol in the Edwards et al. study, effect sizes reported in the current study are small in almost all cases. Key differences which may explain this discrepancy in results include the fact that the study by Edwards et al. delayed vaccination for 6 h after the exercise bout, whereas the current study vaccinated participants immediately (<5 min) post-exercise. Delaying vaccination for several hours after exercise may allow a greater immune response to the exercise bout to develop, although local and systemic immune changes are also noted immediately (Peake et al., 2005). A study aiming to optimize the timing of the bout of eccentric exercise directly compared vaccinating immediately after exercise or delaying for 6 h, but found no difference between any groups in the intervention, including the no-exercise control (Campbell et al., 2010).

A second difference is that the study by Edwards et al. used an exercise intensity of 85% of 1RM for the intervention, which was maintained for all 50 repetitions. The participants in the current study instead were only able to maintain an average intensity of ∼70–75% 1RM across the 50 repetitions. We selected a slightly lower goal intensity at the start of the intervention (80% 1RM) in consideration of joint health of the older, resistance-trained naïve participants and lowered intensity as technique suffered. This intensity is within ranges reported to improve lean muscle mass and muscle strength in older adults following resistance training interventions, thus demonstrating that 65–85% 1RM is safe and induces physiological response in older adults (Peterson et al., 2010, 2011). An alternative strategy could have been to maintain weight but increase intra- and inter-set rest. However, it has been suggested that an adjuvant effect from exercise-induced increases in lymph flow rate are reduced with increased rest periods; this strategy therefore would need careful investigation (Bohn-Goldbaum et al., 2020). Self-reports of muscle soreness were low following our intervention and did not differ in frequency from the No-Ex group, suggesting both that the exercise protocol was well tolerated in this population and that our exercise protocol may not have stimulated a local inflammatory reaction. If so, this would be counter to studies demonstrating leukocytosis and inflammation following acute resistance exercise, even at lower intensities (Nosaka and Newton, 2002; Peake et al., 2005; Szlezak et al., 2016; Chen et al., 2020; Schlagheck et al., 2020). The lack of a systemic marker of inflammation or muscle damage (e.g., creatine kinase) is a limitation to our study. Nevertheless, these differences in intensity may not have influenced antibody titers, as was demonstrated in a direct comparison of the effect of eccentric arm exercise at 60, 85, and 110% of 1RM on antibody responses to influenza vaccination that found no difference between exercise groups (Edwards et al., 2010).

Perhaps the largest difference between the earlier eccentric-exercise investigations and the current study is the fact that our participants were older adults, rather than younger adults who typically achieved clinically protective immune responses to the vaccine even when assigned to the resting control group (Edwards et al., 2007; Campbell et al., 2010; Edwards et al., 2010). In contrast, vaccination of the older participants here largely failed to generate seroconversion, similar to other reports (Goodwin et al., 2006; Van Epps et al., 2017; Bohn-Goldbaum et al., 2020). We had hypothesized that exercise would provide a greater benefit in the current study, as others have found that exercise can enhance immune responses, especially in cases where the control response is weak (Edwards et al., 2007, 2010, 2012; Campbell et al., 2010). Although the exercise effect is not statistically significant, our data offer some support of this idea. There was a trend for an effect of group on fold-change in antibody titer at 6-weeks for B/Colorado/06/2017, apparently due to the larger increase in antibody titer in Ex-S. The B/Colorado/06/2017 strain appeared to be the least immunogenic, as the antibody titers generated to this strain were the lowest on average of the four strains in the vaccine.

The mechanisms underlying exercise-induced improvements in vaccine responses are unknown but may act through the recruitment of immune cells, particularly antigen presenting cells, into local muscle. Eccentric exercise, particularly in those not accustomed to the exercise, results in neutrophil and macrophage infiltration into the exercised muscle which release reactive oxygen and nitrogen species and cytokines (Cannon and St Pierre, 1998; Pizza et al., 2002; Nguyen and Tidball, 2003; Peake et al., 2005). This is similar to the response to chemical adjuvants added to vaccines to enhance immune responses. Like eccentric exercise, chemical adjuvants initiate strong inflammatory reactions and immune cell recruitment at the site of the injection (Mosca et al., 2008; Calabro et al., 2011). Thus, eccentric resistance exercise targeting arm and shoulder muscle may act as an adjuvant through localized inflammation at the site of the vaccine injection. Improvements in antibody-responses to vaccination after cardiorespiratory exercise also suggest that systemic effects of exercise may play a role in immune enhancement (Edwards et al., 2006; Pascoe et al., 2014). Exercise is well known to elicit a transient increase in leukocytes and cytokines in circulation (Walsh et al., 2011). As resistance exercise protocols used to date have primarily included exercise of the arm to be inoculated, it is impossible to separate the local and systemic exercise effects. Comparing cardiorespiratory and resistance exercise protocols is also unsatisfactory, due to difficulties in matching exercise intensity. Although we aimed to overcome these limitations in the current study by including a group matched in exercise but performed in the dominant (and therefore unvaccinated) arm (Ex-Op), the small sample and small effect sizes prevent meaningful comparison. Importantly, both local and systemic immune responses to exercise are reduced in older adults (Simpson et al., 2012). Intensity-matched cardiorespiratory exercise yields a smaller increase in leukocytosis in older adults relative to young adults (Simpson et al., 2012; Spielmann et al., 2014). Although age-related declines in skeletal muscle mass and strength may leave older adults more vulnerable to exercise-induced muscle damage (Manfredi et al., 1991), eccentric exercise-induced increases in circulating and muscle-infiltrating neutrophils and plasma IL-6 are reduced in older adults (Cannon et al., 1994; Toft et al., 2002; Hamada et al., 2005). Thus, it may be that the older adults in the current study did not generate as strong of an immune response to the exercise as younger participants in earlier studies using a similar exercise protocol.

A major limitation of the current study is the small number of participants relative to the effect sizes, which decreased power. The primary reason provided by interested individuals who declined participation was the convenience in receiving their seasonal influenza vaccine from their usual health care provider compared to the time required for participation. This suggests individuals are not opposed to resistance exercise *per se* prior to vaccination, and perhaps recruitment would be higher if it could be performed in the clinic waiting room. An additional reason for screening exclusion was for participation in the prior 6-months in arm resistance exercises. Participants were required to be arm resistance-training naïve to avoid heterogeneity in response due to the repeated-bout effect (Nosaka et al., 2001; Peake et al., 2005) but this did remove a segment of the older adult population that may be most receptive to performing arm resistance exercise prior to vaccination. Further studies are needed to understand if resistance-trained individuals respond differently from resistance-naïve individuals in the effects on vaccination. Additional limitations include the fact that we did not solicit information regarding vaccine reactions other than injection site soreness. A bout of resistance exercise prior to influenza vaccination in older adults has recently been found to decrease vaccine reactions such as fever (Bohn-Goldbaum et al., 2020). Thus, expanding inquiry into vaccine side effects will be important for future research.

## Conclusion

In conclusion, we report that an acute bout of unaccustomed eccentric exercise performed immediately before vaccination with the 2018–2019 quadrivalent influenza vaccine did not yield differences in geometric mean titer, fold-increase, seroconversion, or seroprotection in older men and women, relative to a resting, standard-of-care control. Although the participants were naïve to the exercise, it was well-tolerated. The current study is limited by the small number of participants. Thus, we view these results as providing guidance for future studies targeting the use of exercise as a means of enhancing immune responses to influenza vaccine in older adults.

## Data Availability Statement

The raw data supporting the conclusions of this article will be made available by the authors, without undue reservation.

## Ethics Statement

The studies involving human participants were reviewed and approved by the Institutional Review Board at the University of Houston. The patients/participants provided their written informed consent to participate in this study.

## Author Contributions

EL, RS, and MM designed the study. EL, ME, and MM supervised the study. EL, ME, and ML performed the data analyses. All authors contributed to manuscript writing and approve of the final version to be published.

## Conflict of Interest

The authors declare that the research was conducted in the absence of any commercial or financial relationships that could be construed as a potential conflict of interest.

## Publisher’s Note

All claims expressed in this article are solely those of the authors and do not necessarily represent those of their affiliated organizations, or those of the publisher, the editors and the reviewers. Any product that may be evaluated in this article, or claim that may be made by its manufacturer, is not guaranteed or endorsed by the publisher.
